# Spatial invasion patterns of temporal lobe glioblastoma after complete resection of contrast-enhancing tumor

**DOI:** 10.1007/s11060-025-04991-5

**Published:** 2025-03-05

**Authors:** Jawad Fares, Yizhou Wan, Binay Gurung, Thaaqib Nazar, Richard Mair, Alexis Joannides, Thomas Santarius, Tomasz Matys, Stephen J. Price

**Affiliations:** 1 Cambridge Brain Tumour Imaging Laboratory, Academic Neurosurgery Division, Department of Clinical Neurosciences, University of Cambridge, Cambridge, UK; 2 Academic Neurosurgery Division, Department of Clinical Neurosciences, University of Cambridge, Cambridge, UK; 3 Department of Neurological Surgery, Feinberg School of Medicine, Northwestern University, Chicago, IL USA; 4 Department of Radiology, University of Cambridge, Cambridge, UK

**Keywords:** Glioblastoma, Isocitrate dehydrogenase-wildtype, Temporal lobe, Anterior temporal lobectomy, Supramaximal resection, Magnetic resonance imaging, FLAIR, Diffusion tensor imaging

## Abstract

**Purpose:**

This study investigated invasion patterns of temporal lobe glioblastoma following complete resection of contrast-enhancing tumor and evaluated whether non-enhancing tumor presence in the anterior temporal tip predicts the site of progression.

**Methods:**

We retrospectively analyzed patients from a single-institution database who were diagnosed with IDH-wildtype temporal lobe glioblastoma and underwent complete resection of the contrast-enhancing region. Eligible patients had preoperative, immediate postoperative, and progression MRIs to assess tumor progression patterns. FLAIR imaging was examined for its predictive value in identifying progression sites. Surgical outcomes, progression-free survival (PFS), and overall survival were analyzed.

**Results:**

A total of 48 patients were identified, of whom 14 (29%) underwent anterior temporal lobectomy and were excluded from further analysis. Among the remaining 34 patients, 21 (62%) exhibited anterior progression. Expert assessment suggested that in 12 of these 21 patients (57%), an anterior temporal lobectomy might have encompassed the region of tumor progression. Superior, inferior, and lateral progression patterns were associated with longer median PFS (~ 11 months), whereas medial progression correlated with the shortest PFS (5.9 months). FLAIR signal at the temporal tip had moderate sensitivity (71.43%) but low specificity (18.52%) for predicting anterior progression, resulting in a high false-positive rate.

**Conclusions:**

Temporal lobe glioblastomas often progress anteriorly, suggesting that anterior temporal lobectomy may help reduce progression in select cases. FLAIR imaging had limited predictive value for anterior progression, emphasizing the need for advanced imaging techniques. Future research should identify reliable biomarkers and evaluate the role of anterior temporal lobectomy through well-designed prospective studies.

## Introduction

Glioblastoma, the most common and aggressive primary malignant brain tumor, carries a poor prognosis despite significant advances in surgical and adjuvant therapies. Even with aggressive treatment, including complete resection of contrast-enhancing regions followed by radiotherapy and chemotherapy, the median overall survival remains approximately 16 to 21 months [1, 2]. While maximizing the extent of resection has been consistently associated with improved survival outcomes, local tumor progression remains a frequent and significant challenge, even after complete resection of contrast-enhancing tumor [3].

This persistent challenge has spurred growing interest in supramaximal resections as a strategy to further improve outcomes. One such approach is anterior temporal lobectomy, which involves the removal of the temporal lobe anterior to the tumor, as an alternative strategy for managing temporal lobe glioblastomas [1]. This technique not only addresses the visible tumor but also targets surrounding infiltrative tissues that may harbor microscopic disease. Emerging evidence suggests that anterior temporal lobectomy may enhance survival and seizure control compared to resection limited to contrast-enhancing regions [1, 4–6]. However, the impact of resecting the temporal tip raises a critical question: could this area be a key site for tumor progression in these patients, thereby influencing outcomes?

Despite promising insights, the specific progression patterns of glioblastoma following resection of contrast-enhancing tumor in temporal lobe glioblastomas remain underexplored [3]. Investigating these patterns is essential for refining surgical and radiotherapeutic strategies to improve long-term outcomes.

This study aims to fill this knowledge gap by (1) characterizing invasive progression patterns of temporal lobe glioblastoma after complete resection of contrast-enhancing tumor and (2) assessing whether non-enhancing tumor in the temporal tip predicts progression sites. By elucidating these patterns, we seek to inform optimized, personalized surgical strategies for this aggressive tumor.

## Methods

### Study population

Patients included in this study were identified through a retrospective review of a single-institution tumor database. The cohort consisted of individuals diagnosed with isocitrate dehydrogenase (IDH)-wildtype glioblastoma confined to the temporal lobe who presented for the first time to the neuro-oncology clinic and underwent complete resection of contrast-enhancing tumor between November 17, 2014, and September 12, 2023. All eligible patients received standard postoperative chemoradiotherapy and had magnetic resonance imaging (MRI) data available at three critical time points: preoperative, immediate postoperative, and documented progression. Preoperative imaging was analyzed to assess non-enhancing tumor extension into the temporal tip, while progression imaging documented the spatial trajectory of tumor invasion. This approach enabled evaluation of whether an anterior temporal lobectomy would have encompassed the observed progression area, offering insights into its potential role in managing temporal glioblastoma progression.

Molecular diagnostics, including O6-DNA-methylguanine methyltransferase (MGMT) promoter methylation status and IDH mutation analysis, were retrospectively retrieved from patients’ electronic medical records to complement clinical and imaging data.

### Surgical procedures and assessment

All patients underwent surgical resection guided by 5-aminolevulinic acid (5-ALA) fluorescence. Additional surgical adjuncts, such as neurophysiological monitoring or awake surgery, were utilized at the surgeon’s discretion based on intraoperative needs. The procedures were planned to achieve complete resection of the fluorescent tumor, and in cases involving anterior temporal lobe tumors, the remaining temporal tip was also removed as part of the resection.

Postoperative imaging using T1-enhanced MRI was performed to confirm the absence of residual contrast-enhancing tumor, defining complete resection. Patients were stratified into two groups based on the extent of resection observed on postoperative imaging: those who underwent complete resection of contrast-enhancing tumor without lobectomy and those who underwent resection combined with lobectomy. The latter group was excluded from analysis. To ensure consistency in group classification, an independent neuroradiologist reviewed all MRI scans to verify the completeness of resection.

The assessment of whether an anterior temporal lobectomy might have been beneficial was based on a retrospective review of anatomical imaging, where the criteria included the extent of non-enhancing tumor involvement at the temporal tip, its proximity to critical white matter tracts, and the anticipated feasibility of safely resecting this tissue based on standard neurosurgical anatomical landmarks. The anterior temporal tip was defined as the portion of the temporal lobe located anterior to the contrast-enhancing tumor, typically measuring no more than 4 cm in length.

To ensure a consistent cohort, only tumors confined to the temporal lobe were included in this study, while those extending into other areas, such as the temporal occipital region, were excluded. In cases of anterior progression, we measured the distance between the posterior margin of the temporal tip and the anterior margin of the contrast-enhancing tumor. If this distance was large, indicating that the tumor was located too far posteriorly and that resecting the anterior temporal lobe would require removal of a considerable amount of normal brain tissue, these patients were not considered suitable candidates for anterior temporal lobectomy. This approach ensured that our analysis focused on cases where an extended resection was anatomically feasible.

### Classification of invasion patterns

Tumor progression patterns were classified based on their anatomical relationship to the resection cavity. Anterior progression was defined as tumor extension into the anterior temporal lobe, posterior progression as tumor spread posterior to the resection cavity, and medial progression as invasion into medial temporal structures, including the amygdala and hippocampus. Superior progression was characterized by tumor spread through the temporal stem into the insula or frontal lobe, while distant progression referred to tumor invasion beyond the temporal lobe.

### Statistical analysis

Progression-free survival (PFS) was defined as the time from the initial surgery to the date of documented tumor progression or death from any cause, whichever occurred first. Definitive tumor progression was determined based on radiological criteria, specifically an increase in the size of the lesion on T1-enhanced MRI accompanied by corresponding changes on other sequences (e.g., FLAIR), in line with established guidelines. In cases where imaging findings were ambiguous and raised the possibility of pseudoprogression, patients continued to receive their current treatment regimen. Follow-up imaging was then performed, and only cases where the lesion demonstrated persistent or further radiological enlargement were classified as definitive progression. If subsequent imaging did not confirm progression, the case was categorized as pseudoprogression and not counted as an event for PFS. Patients without evidence of progression or death at the time of analysis were censored at the date of their last follow-up. Overall survival (OS) was defined as the time from the initial surgery to the date of death from any cause or the last follow-up for censored patients. For patients with progression, OS was calculated using the time-to-death data (recorded in days). Median PFS/OS and corresponding 95% confidence intervals for each progression pattern were estimated using non-parametric order statistic methods. For ease of interpretation, all survival times in days were converted to months by approximating 30 days per month.

To assess the relationships between different glioblastoma progression patterns (anterior, posterior, medial, lateral, superior, inferior, and distant), a correlation matrix was generated. Spearman’s rank correlation coefficient was used to evaluate the strength and direction of associations between these progression patterns, as this method accounts for the non-parametric nature of the data.

The diagnostic performance of FLAIR signal into the tip for predicting anterior progression was assessed by calculating sensitivity, specificity, positive predictive value (PPV), and negative predictive value (NPV). Sensitivity was defined as the proportion of true positive cases (patients with FLAIR into the tip and anterior progression) relative to the total number of cases with anterior progression. Specificity was defined as the proportion of true negative cases (patients without FLAIR into the tip and without anterior progression) relative to the total number of cases without anterior progression. PPV and NPV were calculated to determine the proportion of positive and negative test results that were correct, respectively. Confidence intervals for these estimates were calculated using the Wilson method to account for uncertainty in the proportions.

Statistical analyses were performed using IBM SPSS Statistics version 30.0.0, and p-values < 0.05 were considered statistically significant.

## Results

### Patient characteristics

Table 1 summarizes the demographic, tumor, and survival characteristics of patients who underwent complete resection of contrast-enhancing tumors (*n* = 34). The majority of patients were between 50 and 69 years of age (88%, 30/34), with a predominance of males (62%, 21/34). Tumor laterality was evenly distributed. FLAIR signal at the temporal tip was present in 68% of patients (23/34). All cases were IDH wildtype, and MGMT promoter methylation was identified in 32% of patients. The median PFS was 9.67 months, while the median OS was 20.5 months.

**Table 1:** Demographic, tumor characteristics, and survival outcomes for patients undergoing resection of contrast-enhancing temporal lobe glioblastoma

	*N* = 34	Percent
**Age**		
40–49	3	9
50–59	18	53
60–69	12	35
70–79	1	3
**Sex**		
Male	21	62
Female	13	38
**Tumor site**		
Right	19	56
Left	15	44
**FLAIR at the temporal tip**	23	68
**IDH**		
Wildtype	34	100
Mutated	0	0
**MGMT**		
Methylated	11	32
Unmethylated	23	68
**Median PFS (months)**	9.67	
**Median OS (months)**	20.50	

### Invasive patterns

**Fig. 1:**
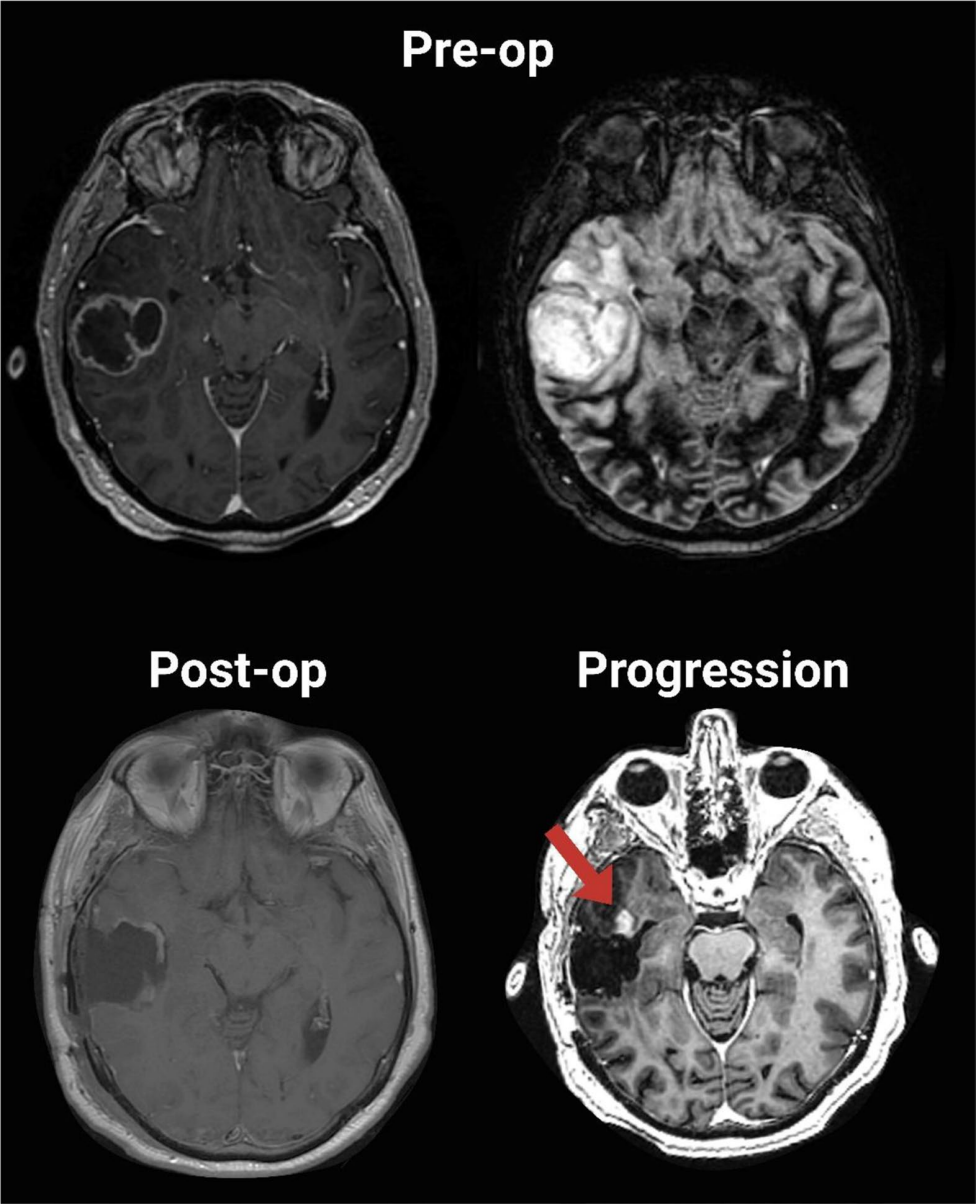
Case example of a 56-year-old female with a right temporal glioblastoma. Non-enhancing tumor extension is observed into the temporal tip. Postoperative imaging confirms complete resection of the enhancing tumor. At progression, initial enhancement appears anterior to the resection cavity (red arrow)

**Fig. 2:**
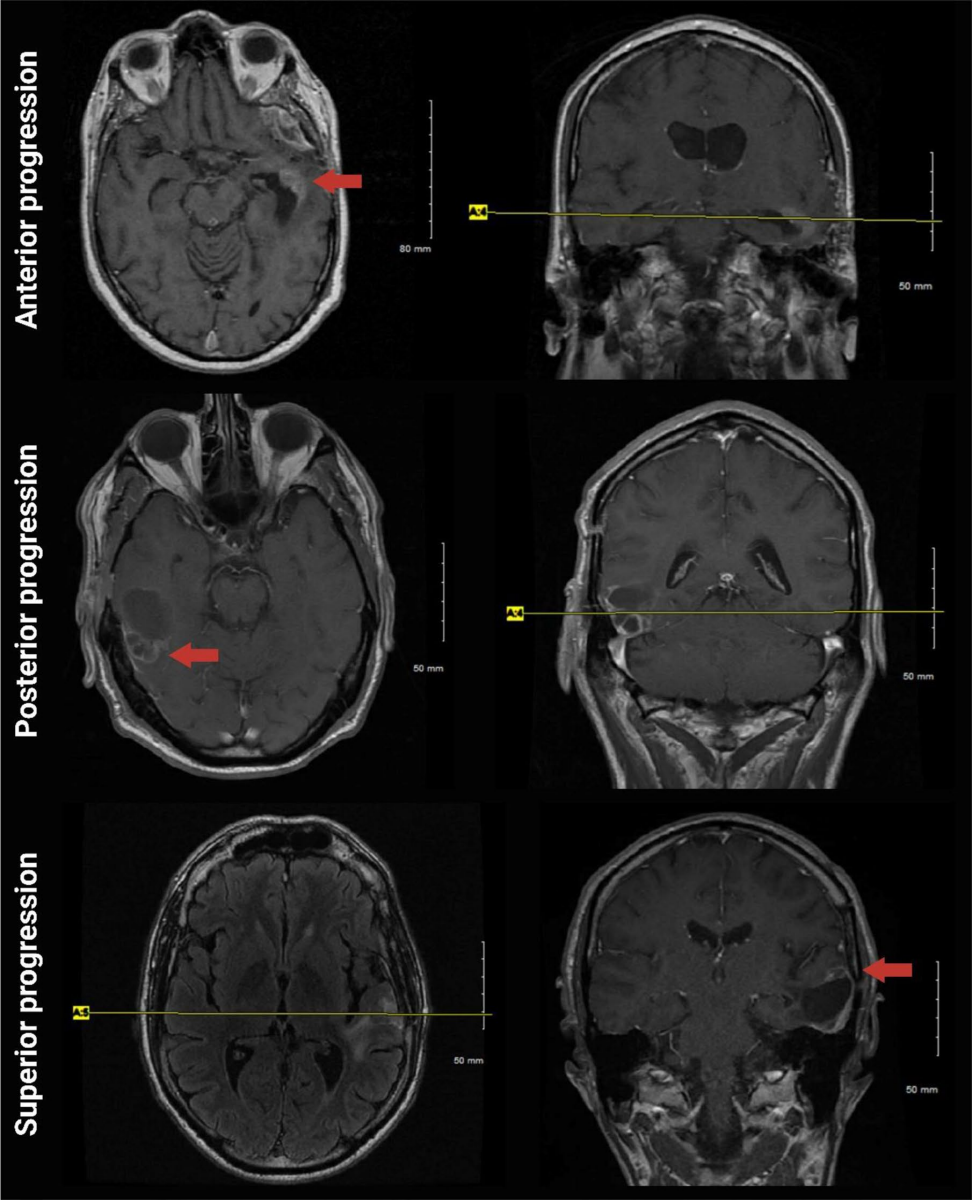
Temporal glioblastoma invasive patterns following gross-total resection. Post-op imaging at progression shows the first enhancement as seen anterior, posterior, or superior to the resection cavity (red arrow)

Table 2 outlines the progression patterns of glioblastoma in the study cohort. The most common progression direction was anterior (62%; Fig. 1), followed by posterior (47%), medial (15%), lateral (12%), superior (12%), inferior (6%), and distant spread (9%) (Fig. 2). Of the 21 patients with anterior progression, nine (43%) exhibited exclusively anterior progression, while the remaining 12 (57%) showed anterior progression along with additional invasion patterns. Correlation matrix analysis revealed weak associations among the various progression patterns, with no statistically significant relationships observed between progression directions.

**Table 2:** Tumor progression patterns following resection of contrast-enhancing temporal lobe glioblastoma

Progression pattern	*N* = 34	Percent
Anterior	21	62
Posterior	16	47
Medial	5	15
Lateral	4	12
Superior	4	12
Inferior	2	6
Distant	3	9

Table 3 summarizes PFS and OS statistics for different tumor invasion patterns after resection. Superior progression exhibited the highest median PFS (11.4 months), followed closely by lateral and inferior progressions (11 months each), while medial progression was associated with the lowest median PFS (5.9 months). Overall survival varied by progression pattern, with median OS ranging from 12.7 months for distant progression to 26.3 months for superior progression; anterior, posterior, medial, lateral, and inferior progressions had median OS values between approximately 20.4 and 21.4 months.

**Table 3:** Progression-free survival (PFS) and overall survival (OS) statistics by tumor progression pattern (*n* = 34)

Progression pattern	Median PFS (months)	95% CI	Median OS (months)	95% CI
Anterior	9.3	6.6–12.3	21	17.0-29.9
Posterior	9.8	6.6–12.4	20.4	16.8–26.1
Medial	5.9	3.3–12.8	20.6	7.1–23.7
Lateral	11	9.4–13.1	20.8	16.4–26.1
Superior	11.4	5.4–28.5	26.3	11.8–61.8
Inferior	11	9.7–12.3	21.4	20.6–22.2
Distant	10.4	8.2–14.9	12.7	11.7–21.5

### Potential predictive biomarkers

Among the 21 patients exhibiting anterior progression, neurosurgeons judged that an anterior temporal lobectomy might have prolonged PFS in 12 cases (57% of those with anterior progression, corresponding to 35% of the overall cohort). An analysis was conducted to evaluate whether FLAIR signal at the temporal tip could serve as a predictive biomarker for anterior progression. FLAIR showed a sensitivity of 71.43% for identifying anterior progression but had a low specificity of 18.52%, indicating a high rate of false positives. The PPV was 40.54%, and the NPV was 45.45%, reflecting limited predictive power. With an overall accuracy of 41.67%.

## Discussion

This study reveals the invasion patterns in patients with temporal lobe glioblastoma after complete resection of contrast-enhancing tumor. Patients most showed anterior progression. PFS varied by direction of invasion. FLAIR signal at the temporal tip had limited predictive accuracy in terms of progression. These results highlight the need for more reliable biomarkers to anticipate invasive patterns and guide surgical decisions effectively.

The anterior spread of temporal glioblastoma may be influenced by the extent of resection and tumor biology. Upon complete resection of contrast-enhancing tumor, non-enhancing tumor tissue at the temporal tip is often left intact, potentially serving as a reservoir for anterior progression [7]. Additionally, resection of contrast-enhancing tumor generally preserves anterior white matter tracts, which glioblastoma cells preferentially invade [8–11], facilitating anterior spread. The limited extent of resection of contrast-enhancing tumor may also maintain components of the local tumor microenvironment, including vascular and stromal support, that further promote anterior progression.

Anterior temporal lobectomy was estimated to be beneficial in 57% of cases with anterior progression. By resecting anterior temporal structures, including the anterior temporal tip, where non-enhancing tumor tissue may reside, and disrupting the anterior white matter tracts that facilitate tumor invasion, this approach could potentially reduce the incidence of anterior progression. Moreover, such an extensive resection may favorably alter the local tumor microenvironment, thereby influencing both tumor invasion and survival outcomes. In this analysis, an expert neurosurgeon reviewed the anatomical extent of tumor spread within the anterior temporal lobe using established neuroanatomical landmarks to assess whether an extended resection might have encompassed the area of progression. This judgment was based on the observed direction of tumor spread. While these retrospective assessments suggest that extended resection may reduce anterior progression, we acknowledge that these conclusions remain speculative and are not supported by direct interventional data.

The variation in PFS based on tumor progression direction may be influenced by anatomical and biological factors that affect tumor spread and treatment response. Lateral and inferior invasion, associated with longer PFS, likely involve regions with fewer critical structures and denser white matter tracts, which may constrain tumor infiltration and slow its advancement [12, 13]. These areas also have better surgical accessibility, allowing for more effective initial resection and reducing the likelihood of aggressive early recurrence. In contrast, medial progression, linked to shorter PFS [14, 15], involves proximity to critical structures such as the thalamus, basal ganglia, and ventricles [16], which not only limits the extent of safe resection but also provides supportive microenvironments that can facilitate faster tumor cell migration and proliferation [17]. Medial regions may also receive higher vascular support, promoting tumor growth and contributing to earlier recurrence [17]. This directional variation in PFS underscores the importance of considering anatomical progression patterns in treatment planning, as these patterns may inherently influence both the aggressiveness of progression and patient outcomes.

The moderate sensitivity but low specificity of FLAIR signal at the temporal tip for predicting anterior tumor spread may stem from the inherent limitations of FLAIR imaging in distinguishing between true tumor infiltration and reactive changes in adjacent brain tissue [18]. FLAIR hyperintensity at the temporal tip is common in patients with temporal lobe glioblastoma due to peritumoral edema, inflammation, or preexisting tissue abnormalities, which can appear similar to non-enhancing tumor invasion on imaging [19–21]. This overlap likely contributes to the high rate of false positives, as FLAIR cannot reliably differentiate infiltrative tumor cells from non-tumorous changes [21, 22]. Additionally, the anterior temporal tip may have a naturally higher prevalence of FLAIR hyperintensity in patients with glioblastoma due to its anatomical susceptibility to tumor-related edema and blood-brain barrier disruption, further decreasing the specificity of FLAIR for predicting true anterior progression. These findings suggest that, while FLAIR can be useful as an initial screening tool, it lacks the precision needed for accurate prognostication and highlights the need for adjunctive imaging modalities, such as diffusion tensor imaging (DTI) or advanced MRI techniques, to better distinguish tumor infiltration from non-specific FLAIR changes.

This study is unique in its focus on detailed progression patterns in temporal lobe glioblastoma, providing insights that may guide tailored surgical approaches. However, several limitations should be noted. Its retrospective, single-institution design may limit generalizability, as patient demographics and imaging protocols could vary in other settings. Additionally, the relatively small sample size (*n* = 34) reduces statistical power and precludes definitive conclusions regarding the potential benefits of more extensive resection strategies. Variability in MRI timing and equipment could also affect consistency in progression assessment. Future prospective, multicenter studies with larger cohorts and advanced imaging techniques are needed to validate and build upon these findings, and to determine whether extended resection of FLAIR-positive tissue can improve survival outcomes.

## Conclusions

This study highlights the invasive patterns of temporal lobe glioblastomas following complete resection of contrast-enhancing tumor and the challenges in predicting progression using conventional imaging. Anterior progression, the most common pattern, may result from limited resection at the temporal tip and preservation of anterior white matter tracts. These findings suggest a potential role for anterior temporal lobectomy in reducing anterior progression through more extensive resection and disruption of invasion pathways. While FLAIR imaging at the temporal tip showed moderate sensitivity, its low specificity limits its predictive utility. Advanced imaging techniques and reliable biomarkers are needed to guide surgical strategies and improve outcomes. Future research should validate these findings and explore innovative, tailored treatment approaches.

## Data Availability

No datasets were generated or analysed during the current study.
